# Neuroimmune interactions: The bridge between inflammatory bowel disease and the gut microbiota

**DOI:** 10.1002/ctm2.70329

**Published:** 2025-05-21

**Authors:** Jinxia Zhai, Yingjie Li, Jiameng Liu, Cong Dai

**Affiliations:** ^1^ Department of Gastroenterology First Affiliated Hospital, China Medical University Shenyang City Liaoning Province China; ^2^ Department of Gastroenterology First Affiliated Hospital, Jinzhou Medical University Jinzhou City Liaoning Province China

**Keywords:** gut microbiota, immune cells, inflammatory bowel disease, neuroimmunology, sensory neurons

## Abstract

**Background:**

The multidimensional regulatory mechanism of the gut–brain–immune axis in the context of inflammatory bowel disease (IBD) has garnered significant attention, particularly regarding how intestinal microbiota finely regulates immune responses through immune cells and sensory neurons.

**Main Body:**

Metabolites produced by intestinal microbiota influence the phenotype switching of immune cells via complex signalling pathways, thereby modulating their anti‐inflammatory and pro‐inflammatory functions during intestinal inflammation. Furthermore, sensory neurons exhibit heightened sensitivity to microbial‐derived signals, which is essential for preserving intestinal balance and controlling pathological inflammation by integrating peripheral environmental signals with local immune responses. The dynamic equilibrium between immune cells and the neuroimmunoregulation mediated by sensory neurons collectively sustains immune homeostasis within the intestine. However, this coordination mechanism is markedly disrupted under the pathological conditions associated with IBD.

**Conclusion:**

An in‐depth exploration of the interactions among immune cells, gut microbiota and sensory neurons may yield significant insights into the pathological mechanisms underlying IBD and guide the creation of new treatment approaches.

**Key points:**

- The gut microbiota regulates the gut‐brain‐immune axis, modulating neuroimmune interactions in IBD.
- Microbiota‐derived metabolites influence immune cells, thereby affecting neurons.
- Neurons secrete mediators, enabling bidirectional neuroimmune communication essential for intestinal homeostasis.
- Disruptions contribute to IBD, offering therapeutic targets.

## Graphical abstract

- The gut microbiota regulates the gut–brain–immune axis, modulating neuroimmune interactions in inflammatory bowel disease (IBD).
- Microbiota‐derived metabolites influence immune cells, driving cytokine release that activates dorsal root ganglion (DRG) neurons.
- DRG neurons secrete mediators, enabling bidirectional neuroimmune communication essential for intestinal homeostasis.
- Disruptions contribute to IBD, offering therapeutic targets. Graphic created with BioRender.com (https://biorender.com).

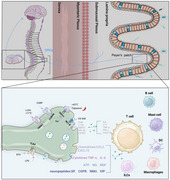

## 1 INTRODUCTION

The gastrointestinal tract, as a crucial immune and neural network, contains 70%–80% of the immune cells in the body, more than 100 million neurons and up to 100 000 extrinsic nerve endings.^1^ The coordinated function of these structures is essential for maintaining host immune balance and regulating inflammation.

Inflammatory bowel disease (IBD) is fundamentally an inflammatory disease, often divided into Crohn's disease (CD) or ulcerative colitis (UC) using clinical, endoscopic and histopathological criteria.^2^ CD and UC differ in many aspects, encompassing affected areas, types of immune responses, neuronal counts, neurogenesis and the roles of glial cells (Table 1). The occurrence of IBD results from genetic susceptibility, gut microbiota imbalance and environmental influences. Dysregulated gut microbiota can activate the adaptive immune system, fostering inflammatory responses and leading to chronic inflammation and tissue injury. Immune cells are crucial in the gut's immune response, and their aberrant activation is a hallmark of IBD.

**TABLE 1:** Crohn's disease (CD) and ulcerative colitis (UC) differ in many aspects in inflammatory bowel disease (IBD).^147^

	UC	CD
Affected area	Limited to the colon	Primarily affects the distal small intestine and colon
Type of immune response	Type 2 immune response	Type 1 or Type 17 immune response
Major cytokines	IL‐5, IL‐13	IL‐12, IL‐17, IL‐23, IFN‐γ, TGF‐β
Enteric nervous system (ENS) function	Significant differences in ENS function among patients	Influenced by genetics, diet, microbiota and infection history
Role of glial cells	Less studied	Glial cells release CSF, activate macrophages and promote inflammation
Neurogenesis	5‐HT4‐dependent pathways; differentiation of Sox2+ glial cells into new enteric neurons	ATP via P2X7 receptor, Panx1 and caspases causes neuron death
Neuron count	Increased neuron count (associated with *Clostridium difficile* infection)	Decreased neuron count or no significant change, varies with disease severity

The gut microbiota interacts with immune cells through its metabolic products, and it also activates peripheral sensory neurons, mainly dorsal root ganglion (DRG) neurons and nodose vagal ganglia neurons. These interactions jointly regulate intestinal function and significantly impact inflammation and host defence mechanisms. In IBD, the microbiota modulates the inflammatory and anti‐inflammatory roles of T cells, thereby influencing immune dynamics in the gut. Moreover, these peripheral sensory neurons rapidly detect microbial signals and modulate gut immune responses through neuroimmune interactions, highlighting their key role in the development of IBD. The relationship between the immune system and the peripheral sensory nervous system is critical in regulating gut inflammation. These interactions create a complex communication network through bidirectional signalling via neurotransmitters, neuromodulators and cytokines. Studies suggest that these networks are vital for keeping gut immune balance and protecting against pathogens.

Recent research has deepened our comprehension of the gut–brain–immune axis in neurobiology and immunology, particularly the complex interactions among the gut microbiota, the immune system and peripheral sensory neurons. This review will examine the interactions among gut microbiota, immune cells and peripheral sensory neurons in IBD, focusing on how the microbiota influences immune balance and inflammatory responses through the coordinated actions of these systems. To ensure the comprehensiveness of this review, a systematic literature search was conducted using PubMed, Cochrane CENTRAL, Medline, Ovid Embase, along with conference abstracts such as Colitis Organization (ECCO), European Crohn's, United European Gastroenterology (UEG) week and Digestive Disease Week (DDW). With keywords including ‘neuroimmune interactions’, ‘intestinal microbiota’, ‘Crohn's disease’, ‘Ulcerative colitis’, ‘IBD’. The search covered studies published between 2000 and 2024, focusing on articles and excluding non‐English publications.

## 2 THE ROLE OF NEUROIMMUNE INTERACTION IN IBD

### 2.1 Regulation of intestinal function by the nervous system

Intestinal function is controlled by the nervous system via both the peripheral nervous system and the central nervous system (CNS). Regulation within the peripheral nervous system is primarily achieved via the autonomic and sensory components.^3^, ^4^ The autonomic nervous system encompasses the sympathetic, parasympathetic and enteric nervous systems (ENS). Notably, the ENS's sensory component, which consists of intrinsic primary afferent neurons, establishes a comprehensive reflex circuit with enteric interneurons and motor neurons, thereby facilitating intestinal function regulation.^5^

The spinal cord and vagal sensory afferent systems innervate the intestines and transmit noxious stimuli. Their cell bodies reside in the DRGs and vagal ganglion, respectively. The terminals of spinal afferent nerves terminate in the dorsal horn of the spinal cord, whereas vagal afferent nerves project to the nucleus of the solitary tract in the brainstem. These nerves innervate the muscular and mucosal layers of the intestine and are involved in controlling the immune response within this organ.^6^ Notably, vagal innervation is most densely concentrated in the proximal small intestine, with a reduction observed in the large intestinal wall. Spinal afferent nerves from thoracolumbar DRG predominantly supply the small intestine, while those from lumbosacral DRG mainly target the large intestine. Nociceptors, which are specialised sensory neurons, store neuropeptide vesicles in the terminals of both central and peripheral tissues, and they are crucial for the perception of harmful external stimuli as well as the regulation of immune responses.

The adaptability of enteric neurons to changes in their microenvironment, known as enteric neuronal plasticity, represents a key response mechanism to diverse pathological stimuli.^7^ These microenvironments include intestinal microbiota and their metabolites,^8^ intestinal glia,^9^ sex hormones,^10^, ^11^ a high‐fat diet,^12^ hyperglycaemia^13^ and various intestinal and neurological diseases.^14^ Importantly, early psychological stress can also affect enteric neurons.^15^, ^16^ Studies have demonstrated that chronically elevated glucocorticoid levels during stress induces an inflammatory subpopulation of enteric glia cells, enhancing monocyte‐ and TNF‐driven inflammation through CSF1. Glucocorticoids also trigger transcriptional immaturity in enteric neurons, resulting in acetylcholine deficiency and dyskinesia through TGF‐β2, thereby influencing the progression of IBD.^16^ Additionally, research indicates that sustained stress can trigger autophagy in intestinal dopaminergic neurons, contributing to gastrointestinal motility dysfunction.^17^ Increased corticosteroidemia resulting from early life adversity may be associated with ENS remodelling, which reduces autophagy levels in enteric neurons. The inhibition of autophagy renders enteric neurons more susceptible to cellular stress.^18^ Notably, this finding contrasts with previous reports suggesting that stress induces autophagy in intestinal dopaminergic neurons, thereby affecting intestinal function, while other studies suggest that stress reduces autophagy in enteric neurons, consequently impacting gut function. This discrepancy may arise from the differential effects of various types of stress or different temporal windows on autophagy, or it may be due to the distinct autophagy mechanisms in different types of intestinal tract neurons. The role of neuronal autophagy in the intestine during disease warrants further investigation. Damage to enteric neurons is a critical factor in gastrointestinal symptoms experienced by IBD patients. Therefore, targeting environmental factors that harm enteric neurons could greatly impact IBD treatment.

### 2.2 The role of the enteric nervous system in intestinal inflammation

ENS plays a crucial role in the development, maintenance and regulation of intestinal inflammation. Comprising a highly interconnected network of neurons and enteric glial cells (EGCs), the ENS regulates gut homeostasis and inflammatory responses through complex neuroimmune–microbiota interactions. Research has shown that the severity of intestinal inflammation correlates with the density of neural innervation, with a greater number of neurons associated with more pronounced inflammatory responses.^19^ Emerging evidence highlights the context‐dependent duality of ENS‐mediated neuroimmune regulation, particularly through its interaction with CD8+ T cells. Experimental models demonstrate that EGCs can activate CD8+ T cells via major histocompatibility complex class I (MHC I) presentation during *Toxoplasma gondii* infection, triggering IFN‐γ and TNF production to enhance anti‐pathogen immunity.^20^ Paradoxically, in non‐infectious inflammation such as IBD, neuronal MHC I‐mediated CD8+ T cell activation drives neuronal loss and barrier dysfunction through excessive inflammation.^21^ This functional dichotomy of MHC I signalling between infectious and sterile inflammation underscores its critical role in determining inflammatory outcomes. Controversies persist regarding EGC antigen presentation capabilities. While IBD patient biopsies show MHC II expression in ileal^22^ and colonic^23^ EGCs, DSS colitis models demonstrate MHC I but not MHC II expression in EGCs.^20^ This discrepancy may arise from methodological limitations, including macrophage contamination or morphological misidentification. Importantly, functional studies confirm EGCs lack classical MHC II‐mediated antigen presentation. These findings redefine EGCs as immunomodulatory ‘border‐crossing’ cells rather than mere neural support elements, with MHC I‐mediated CD8+ T cell regulation constituting a core anti‐infective mechanism, while MHC II expression likely reflects inflammatory status rather than functional antigen presentation. The ENS further modulates intestinal inflammation through neurotransmitter signalling. 5‐HT exemplifies this dual regulatory capacity: 5‐HT4 receptor activation promotes neuronal survival and epithelial repair via PKA/ERK pathways,^24^ while 5‐HTR4 and 5‐HTR2A stimulation enhances epithelial proliferation and differentiation.^25^ Conversely, chronic inflammation induces pathogenic 5‐HT3‐mediated signalling, amplifying nociceptive transmission to the CNS and promoting pro‐inflammatory cytokine release (TNF‐α, IL‐1β, IL‐6) that exacerbates Th1‐dominant responses and visceral hypersensitivity.^26^ This neuroimmune plasticity highlights the ENS's dynamic adaptability to inflammatory milieus. Notably, the ENS mediates stress‐aggravated inflammation in IBD. Chronic glucocorticoid elevation induces a pro‐inflammatory EGC subtype that secretes CSF1 to drive monocyte‐derived TNF production, while TGFβ2‐mediated transcriptional immaturity in enteric neurons disrupts intestinal motility.^16^, ^27^ Additionally, ENS‐regulated luminal pH modulation emerges as a critical mechanism shaping microbial communities and inflammatory processes.^28^ In conclusion, the ENS exerts multifaceted control over intestinal inflammation through MHC I‐mediated CD8+ T cell activation, neurotransmitter signalling (particularly 5‐HT pathways), stress signal transduction and luminal microenvironment regulation. The controversial antigen‐presenting role of EGCs appears context‐dependent, varying across inflammation types and experimental models. Future investigations should prioritise elucidating context‐specific neuroimmune interaction mechanisms to identify novel therapeutic targets for IBD and infectious enteropathies.

### 2.3 Dual roles of neurons in IBD

Human sensory neurons comprise diverse subtypes responsible for mediating sensations such as heat, cold, pain and itch. In recent years, the scientific community has increasingly focused on the role of peripheral sensory neurons in IBD. Their role in IBD is increasingly acknowledged to encompass both detrimental and protective effects. Suppression of sensory nerves through physical denervation or chemical ablation may not only exacerbate inflammation,^29^, ^30^ such as by increasing the production of pro‐inflammatory cytokines, but may also alleviate colon inflammation in models of dextran sodium sulphate and 2,4,6‐trinitrobenzenesulphonic acid‐induced colitis.^31^

In IBD, transient receptor potential (TRP) channels are implicated in abdominal pain in experimental IBD models. Specific ion channels on sensory neurons, such as TRPA1, TRPV1 and TRPM8, as well as neuropeptides released by these neurons—including substance P, calcitonin gene‐related peptide (CGRP), vasoactive intestinal peptide and pituitary adenylate cyclase‐activating polypeptide—play distinct roles in the pathophysiology of IBD. Among these, in mouse models, TRPM3 plays a role in colonic sensory signalling and could be a potential target for relieving IBD‐associated pain.^32^ TRPV1 is primarily expressed in DRG^33^ and nodose ganglia, where it plays a key role in nociception and inflammatory responses.^34^ TRPV1 mediates mechanical and thermal hyperalgesia, while the release of CGRP exerts anti‐inflammatory effects. TRPA1, involved in cold‐induced pain, has a dual role in mice with colitis.^35^, ^36^ Meanwhile, TRPM8 has been shown to exert a protective effect in 2,4,6‐trinitrobenzenesulphonic acid‐induced colitis in mice.^37^ While the expression of TRPM2 and TRPV2 is increased in the colon of the 2,4,6‐trinitrobenzenesulphonic acid‐induced IBD rat model, oral administration of econazole (TRPM2 inhibitor)^38^ and TRPV2 inhibitor^39^ helps to reduce visceral hypersensitivity. Similarly, inhibiting TRPV4 expression in mouse models may also help treat IBD.^40^ CGRP is a neuropeptide that functions as a bidirectional neuroimmune modulator, altering the functions of immune cells. Various immune cells, including monocytes, B cells and T cells, interact with sensory neurons by releasing CGRP.^41^ CGRP has been found to mitigate 2,4,6‐trinitrobenzenesulphonic acid and dextran sodium sulphate‐induced colitis in rats.^42^ Additionally, substance P, vasoactive intestinal peptide and pituitary adenylate cyclase‐activating polypeptide play key roles in promoting tissue repair and regulating immune responses, exerting both anti‐inflammatory and pro‐inflammatory effects depending on the context of the inflammatory process.^43^ In IBD patients, the epithelial barrier is damaged,^44^ and nerves can also affect the disease by regulating epithelial barrier function and pain. For instance, TNF‐α weakens intestinal barrier function, and the muscarinic M3 receptor increases the activity of TNF‐α converting enzyme by activating p38 MAPK, promoting the shedding of TNF receptors and inhibiting the effects of TNF‐α. TNF‐α converting enzyme also activates the epidermal growth factor receptor, which helps maintain epithelial barrier function.^45^ IFN‐γ inhibits ERK and FAK signalling mediated by M1 muscarinic acetylcholine receptors, thereby compromising barrier integrity. Interestingly, the increased phosphorylation of ERK and FAK observed in IBD patients may represent a compensatory response.^46^, ^47^, ^48^, ^49^ In mouse models, sensory neurons release CGRP, which modulates microfold cell density and segmented filamentous bacteria levels, contributing to barrier protection and reducing pathogen infection risks.^50^ Additionally, engineered *Bacteroides* strains that produce sustained levels of tryptamine stimulate mucus secretion by goblet cells via 5‐HT4 receptors, alleviating IBD severity and preserving barrier integrity in mice.^51^ The cholinergic nervous system also plays a key role in anti‐inflammatory responses and barrier protection. For example, in rats, carbachol acts through the α7 nicotinic acetylcholine receptor to inhibit NF‐κB and MLCK signalling pathways, mitigating tight junction damage and restoring barrier function.^52^ In IBD, inflammatory factors such as IL‐1β, TNFα, IL‐6 and cysteinyl leukotrienes modulate pain responses by affecting the activity of DRG neurons. TNFα, in particular, regulates K(v) and Na(v) currents through TNF receptors on DRG neurons, playing a role in pain modulation mechanisms in mice.^53^ The role of the sympathetic nervous system in IBD is dual‐faceted, as it can both promote and mitigate inflammation. In contrast, the vagus nerve significantly influences IBD by regulating immune responses through different acetylcholine receptor subunits.^54^ Notably, vagus nerve stimulation has shown promising therapeutic effects in patients with CD.^55^ Ongoing clinical trials indicate that stimulating the autonomic nervous system can significantly improve barrier function and alleviate disease symptoms.^56^

These data suggest that neurons exert various effects on IBD through multiple mechanisms, including the involvement of TRP channels and sodium–potassium channels on the cell membrane, the release of neuropeptides and the regulation of the epithelial barrier. In the inflammatory response of IBD, what is the impact of immune cells on IBD? Exploring their role could pave the way for novel IBD treatments.

### 2.4 Dual role of immune cells in IBD

Immune cells play a dual function in IBD. While they can trigger chronic inflammation and tissue damage, they are also essential for maintaining the intestinal barrier, promoting host defence and resolving inflammation. Innate immunity is the first line of defence against microbes, leading to the activation of adaptive immunity.^57^ Activated T lymphocytes infiltrate the mucosa and target epithelial cells, intensifying inflammation.^58^ Different subtypes of T cells are differentially expressed and have different functions in IBD. Studies have found that patients with UC have an increase in IL17A^+^ CD161^+^ effector memory T cells, IL17A^+^ Treg and CD8^+^ TRM cells,^2^ and patients with CD have an increase in IL1B^+^ HLA‐DR^+^ CD38^+^ T cells.^59^ An increase in Th17 cells is linked to susceptibility to inflammatory diseases.^60^ In mouse models colonised with microbiota from IBD patients, Th17 cells are elevated.^61^ In mouse models, certain microbes, such as *segmented filamentous bacteria*,^62^ *Bifidobacterium adolescentis* ^63^ and *Escherichia coli*,^64^ are known to induce Th17 cells. Reducing these Th17‐inducing pathogenic microbes may help alleviate IBD symptoms. These abnormal T cell activations are linked to intestinal barrier disruption, epithelial cell apoptosis and loss of immune tolerance, driving chronic inflammation and tissue damage.

Although immune cells possess pro‐inflammatory and destructive capacities, they are essential for maintaining gut health. For example, during the pathogenesis of IBD, Tregs suppress overactive immune responses by secreting anti‐inflammatory cytokines and protecting the intestinal mucosa from further damage. However, the function of Treg cells is usually suppressed in IBD patients, and studies have found that ZEB2 gene activity is increased in Treg cells of UC patients, which weakens the regulatory ability of Treg. Faecal transplantation in IBD patients reduces RORγt^+^ Treg cells.^65^ Which provide a defensive effect on the disease in IBD mouse models.^66^, ^67^, ^68^ In addition, CD4^+^Foxp3^+^Treg cells also play a key function in the pathogenesis of IBD by inhibiting inflammation.

In addition to T cells, macrophage^69^ and mast cells^70^ also play crucial roles in the pathogenesis of IBD. Macrophages are not merely ‘scavengers’; they perform different roles in various intestinal environments, which are determined by the surrounding cells and microenvironments. Macrophages in the lamina propria can directly sense signals from invading bacteria, playing a pro‐inflammatory role.^71^ Although macrophages in the submucosal layer, particularly those in the muscularis, are less likely to directly sense disturbances from the lumen, in the muscularis, the abundant distribution of norepinephrine nerve fibres forms synapses with macrophages in this region.^72^, ^73^ α‐Adrenergic signalling enhances irritation, while β‐adrenergic signalling suppresses both innate and adaptive defences.^74^ Macrophages express high amounts of β2‐adrenergic receptors (β2AR),^75^ and the norepinephrine released by nerve fibres targets these β2AR^+^ macrophages, thereby alleviating the pro‐inflammatory state induced by macrophages in this area. This is in contrast to macrophages oriented along the serosa and longitudinal muscle. These findings suggest that macrophages in different regions of the intestine have distinct roles in disease pathogenesis, maintaining the balance between anti‐inflammatory and pro‐inflammatory responses. Through neuroimmune communication between macrophages and enteric neurons, a rapid tissue‐protective response can be initiated when the intestine is disturbed from distal sites.^76^ Analysing macrophage function itself, and how they interact with surrounding cells, such as epithelial cells, neurons and endothelial cells, will help us better understand the pathogenesis of IBD. In IBD, mast cells may also participate in neuroimmune interactions that lead to visceral sensitivity and motility disturbances. Studies suggest that LMIR3‐deficient colonic mast cells play a role in exacerbating colitis induced by dextran sulphate sodium, which is linked to mast cell activation‐mediated intestinal inflammation.^63^ Psychological stress is known to elevate intestinal permeability by activating mast cells via corticotropin‐releasing hormone, further affecting IBD.^77^ In a chronic colitis rabbit model, mast cells mediate the inhibition of NaCl absorption through suppression of Cl: HCO3 exchange, which is a major cause of diarrhoea in IBD patients.^78^ Mast cell stabilisers, such as ketotifen,^79^, ^80^, ^81^ and protease inhibitors like APC2059,^82^ have already been proven effective in clinical trials for symptom management in IBD patients.

In summary, immune cells are not only key regulators of intestinal inflammation but also important maintainers of intestinal barrier homeostasis. Their abnormal activation and immune dysregulation are one of the core mechanisms of chronic inflammation in IBD. In this context, understanding how sensory neurons interact with immune cells and understanding the immune cell‐sensory neuron crosstalk in IBD may open new avenues for treating inflammation and pain in IBD.

### 2.5 The neuroimmune interaction

The immune systems and nervous communicate primarily through shared signalling molecules such as neuropeptides, cytokines and chemokines, working together in response to environmental stimuli.^6^, ^83^, ^84^ For example, immune cells release mediators that can interact with neuronal axons via pattern recognition receptors (like Toll‐like receptors, TLRs), cytokine receptors (such as IL‐1β and TNF‐α) and lipid mediators receptors. These interactions activate neurons, leading to action potential generation or regulating neuronal activity through anterograde or retrograde axonal transport. This sensitisation of peripheral sensory neurons may result in changes in spinal cord and brain function, contributing to chronic pain.

Conversely, neurons influence immune cell activity by secreting neurotransmitters like catecholamines, gamma‐aminobutyric acid, acetylcholine and neuropeptides like CGRP, substance P and neuromedin U, which bind to receptors on immune cells.^85^, ^86^ This bidirectional communication maintains the balance between the nervous and immune systems and forms the basis for their mutual regulation in inflammation and pathology (Graphical abstract).

#### 2.5.1 Distribution of immune cells and sensory neurons in the intestine

In recent years, the molecular mechanisms of bidirectional neuroimmune signalling have become a key research focus. There are three types of sensory neurons that provide innervation to the gastrointestinal tract: DRG neurons, nodose ganglia neurons and intrinsic primary afferent neurons. Studies show that various immune cell populations—such as macrophages, mast cells, dendritic cells, CD4^+^ T cells, γδ T cells and innate lymphoid cells—are often located near these sensory neurons, forming neuroimmune cell units. For example, T cell zones and the dome region of Peyer's patches are densely innervated, with some nerve fibres extending into follicles to directly contact B220^+^ B cells, CD3^+^ T cells^87^ and IgA‐producing plasma cells.^88^ CGRP^+^ and NOS1^+^ nerve fibres are co‐localised with Foxp3^+^ Treg cells, suggesting possible interactions between these fibres and Tregs.^89^ These neuroimmune units are regulated by the gut microbiota^90^ and influence intestinal homeostasis through local signalling and bidirectional communication along the neuroimmune axis.

#### 2.5.2 The impact of neurons on immune cells

**FIGURE 1:**
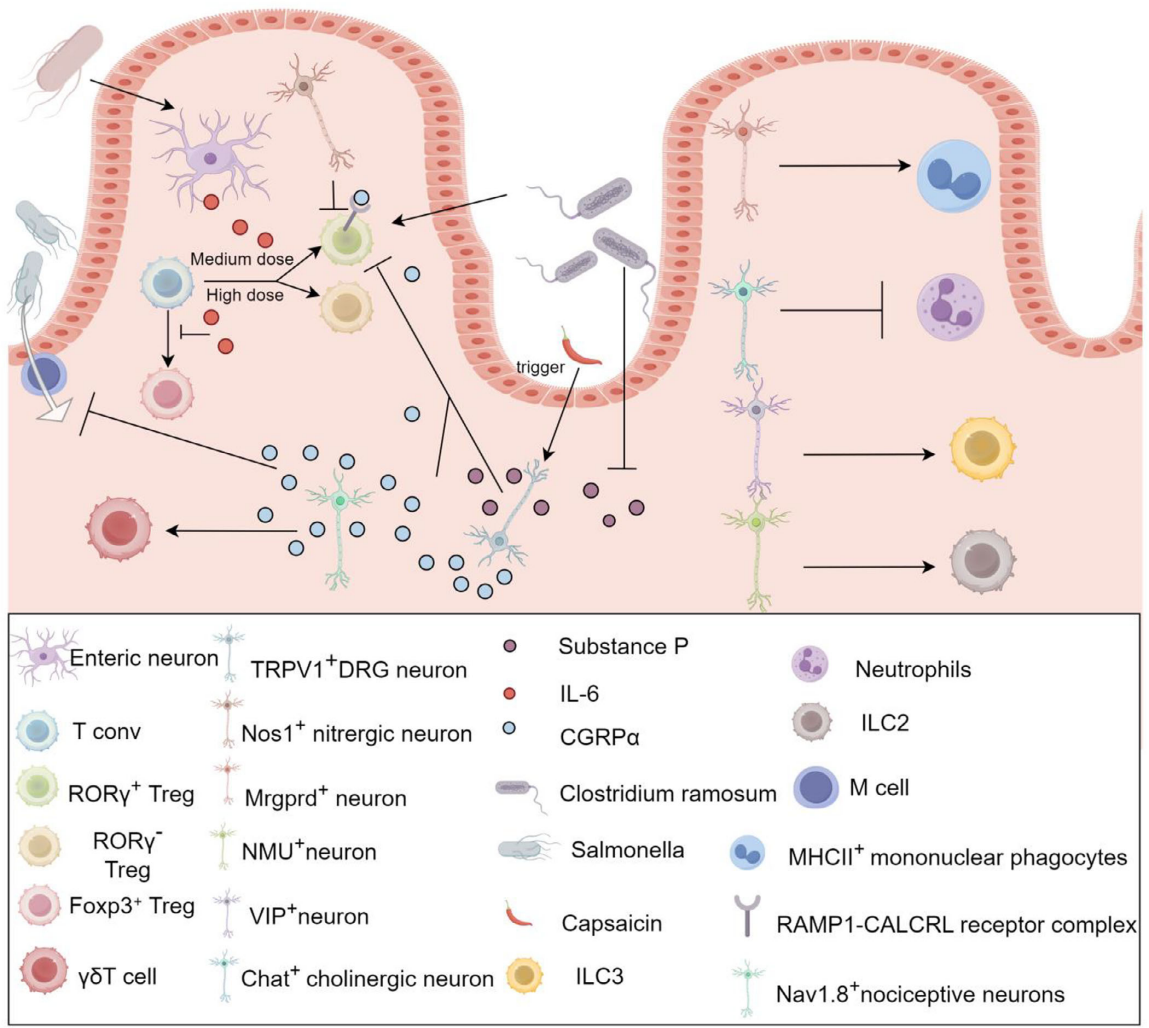
Neuronal regulation of immune cell activation. (In the gut, the intestinal microbiota influences T cell differentiation by activating the enteric nervous systems [ENS] to release IL‐6, with moderate IL‐6 levels promoting differentiation into RORγ⁺ Tregs, while high IL‐6 levels favour differentiation into RORγ⁻ Tregs. Nos1⁺ neurons inhibit the differentiation of RORγ⁺ Tregs. Capsaicin and *Clostridium ramosum* activate TRPV1⁺ neurons, triggering the release of substance P, which suppresses the differentiation of RORγ⁺ Tregs. Meanwhile, TRPV1⁺ neurons release CGRPα, which binds to the RAMP1‐CALCRL receptor complex on RORγ⁺ Tregs, negatively regulating their differentiation. Additionally, TRPV1⁺ and Nav1.8⁺ neurons release CGRPα, which inhibits M cell development in Peyer's patches, thereby preventing *Salmonella* invasion. Nav1.8⁺ neurons also release CGRPα to activate γδT cells. This figure was created using Figdraw (www.figdraw.com)).

The bidirectional interaction between immune cells and neurons is gradually being elucidated. First, neurons significantly influence immune cell function. Research indicates that sensory fibres modulate the activation state of T cells, with T cells located near sensory fibres exhibiting lower levels of activation, while those farther away display higher activation levels.^91^ At the same time, ENS can regulate the differentiation of Tregs by secreting IL‐6.^89^ Activation of Nos1^+^ neurons decreases the number of RORγ^+^ CD4^+^ T cells (TH17‐like) in the ileum of Nos1^−^ADC mice, whereas activation of Chat^+^ cholinergic neurons reduces the number of neutrophils in the ileum. In contrast, activation of Mrgprd^+^ neurons increases the number of MHCII^+^ macrophages in the ileum. Additionally, neurons expressing vasoactive intestinal peptides influence immune responses by regulating the activity of innate lymphoid cells type 3, while gut neurons expressing neuromedin U amplify innate lymphoid cells type 2‐mediated immune responses, thereby enhancing immune activity. Activation of TRPV1^+^ DRG reduces the numbers of RORγ^+^ Treg cells and macrophages in the cecum and colon, through the release of CGRPα, which binds to the RAMP1‐CALCRL receptor complex on RORγ^+^ Treg cells, exerting a negative regulatory effect,^92^ further experiments suggest that capsaicin may directly or indirectly inhibit the differentiation of colonic Tregs into RORγ⁺ Tregs by inducing the activation of TRPV1⁺ sensory neurons in the gut and promoting the release of substance P.^93^ The bacterium *Clostridium ramosum* has been shown to negatively regulate the expression of substance P^93^ in the gut while simultaneously inducing the differentiation of RORγ⁺ Tregs in the colon,^66^ further supporting the notion that TRPV1⁺ neurons suppress the differentiation of RORγ⁺ Treg cells through the release of both CGRPα and substance P. Additionally, Nav1.8⁺ nociceptive neurons activate γδT cells via the retrograde release of CGRP,^94^, ^95^ and both TRPV1⁺ and Nav1.8⁺ neurons regulate immune responses by releasing CGRP, which in turn inhibits the development of M cells in Peyer's patches, thereby limiting Salmonella invasion.^50^ These findings highlight the pivotal role of neuronal activity in dynamically shaping immune responses through spatial gradient regulation (wherein immune responses are modulated based on the physical proximity between T cells and neurons), cell subtype‐specific interactions (such as the regulatory influence of Nos1⁺, Mrgprd⁺ and VIPergic neurons on distinct immune cell populations), and molecular signalling pathways (including the CGRP‐RAMP1/CALCRL axis), which governs neuroimmune communication. Figure 1 reveals that different types of neuronal cells play important roles in the functions and differentiation of different immune cells during homeostasis and intestinal infections. The growing body of evidence underscores the central role of neuronal activity in immune cell activation and immune regulation, offering new insights into the intricate and dynamic interplay between the nervous and immune systems.

#### 2.5.3 The impact of immune cells on neuron function

Conversely, immune cells also influence neuronal function through various mechanisms. During innate immune responses, *Yersinia pseudotuberculosis* and the helminth *Strongyloides venezuelensis* engage in neuroimmune interactions to mitigate infection‐induced neuronal loss. *Y. pseudotuberculosis* activates sympathetic neurons, triggering the release of norepinephrine, while *S. venezuelensis* induces eosinophils to secrete IL‐4 and IL‐13. These signalling molecules act on β₂‐adrenergic receptors on muscularis macrophages, prompting the release of arginase‐1, which exerts a neuroprotective effect by reducing inflammation‐driven neuronal damage. This underscores the importance of host‐microbe interactions in shaping the gut's immune‐neuronal landscape.^96^ Conversely, certain immune responses contribute to neuronal damage and dysfunction. In West Nile virus (WNV) infection, infiltration of CD8⁺ T cells into the intestinal mucosa leads to the targeted destruction of enteric neurons, impairing gut motility.^97^ Similarly, in IBD, CD8⁺ T cells accumulate in the submucosal and myenteric plexuses. IFN‐γ further exacerbates neuronal vulnerability by inducing MHC I expression on enteric neurons, making them more susceptible to CD8⁺ T‐cell‐mediated cytotoxicity.^98^ This process ultimately disrupts ENS integrity and significantly impairs gut motility, reinforcing the notion that chronic inflammation can drive functional neurodegeneration in the gut. Beyond their role in neuronal survival and inflammation, immune cells actively shape neuronal signalling and sensory perception. CD4⁺ T cells promote the synthesis and secretion of β‐endorphins by enteric neurons, thereby modulating pain thresholds and contributing to endogenous analgesic mechanisms.^99^ Additionally, B cells influence neuronal activity by secreting IgE, which binds to FcεRI receptors on enteric neurons, triggering the release of adenosine. Adenosine then engages A3 receptors on mast cells, amplifying their activation and establishing a neuroimmune positive‐feedback loop.^100^ Trypsin (chymase), a major component in mast cell granules, can bind to the protease‐activated receptor 1 on TRPV1^+^DRG neurons, thereby activating the neuron,^101^ activation of protease‐activated receptor 1^102^ and protease‐activated receptor 2^103^, ^104^, ^105^ receptors has been shown to activate the PKC pathway, leading to phosphorylation of the TRPV1 receptor, which in turn leads to channel opening.

**FIGURE 2:**
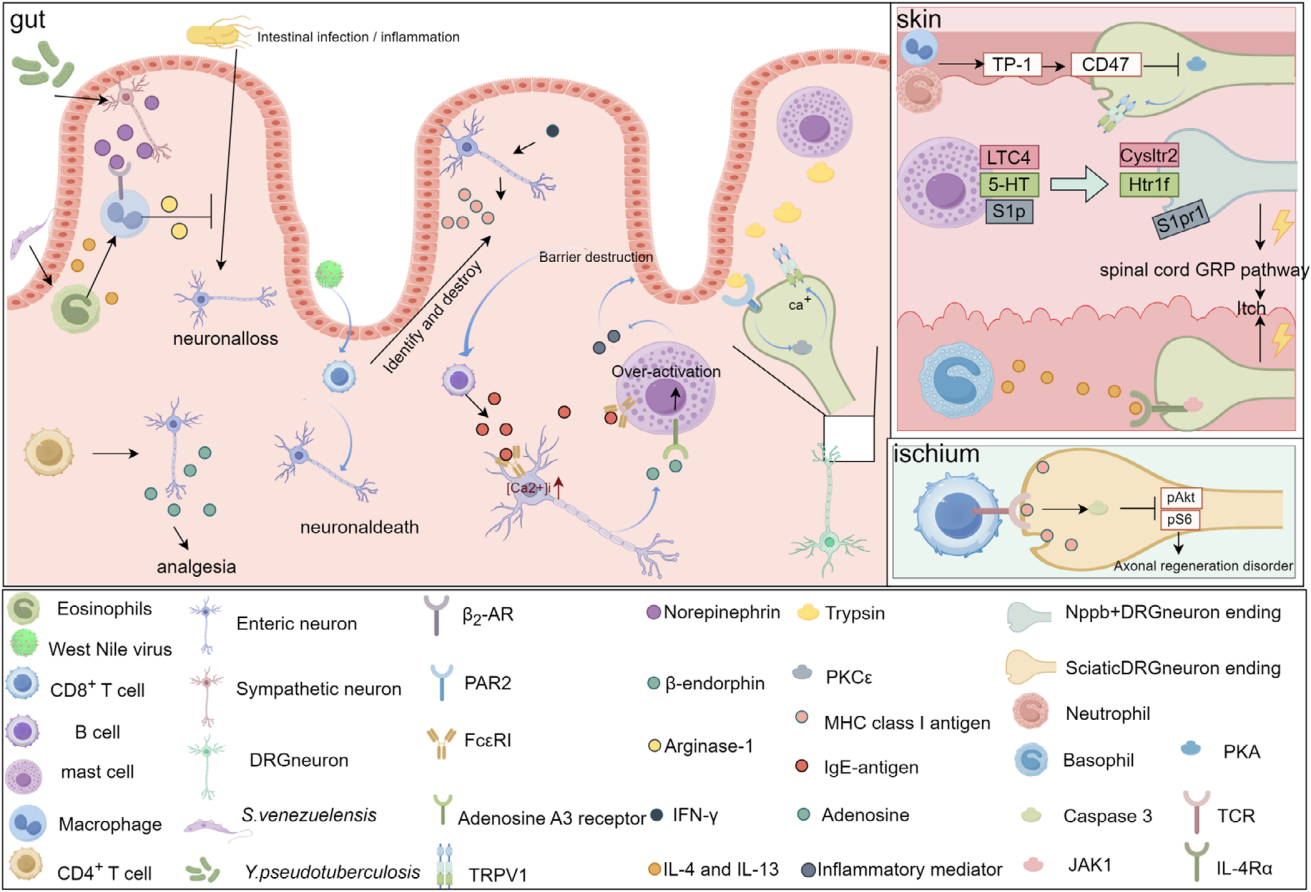
Immune cells modulating neuronal function. (In the gut, various immune cells and pathogens interact to influence neuronal function. *Yersinia pseudotuberculosis* stimulates sympathetic neurons to release epinephrine, which binds to β₂‐AR on macrophages, leading to their activation and subsequent release of arginase, thereby preventing inflammation‐induced neuronal loss. Similarly, *Strongyloides venezuelensis* infection promotes eosinophils to release IL‐4 and IL‐13, which also activate macrophages and enhance arginase production, providing neuroprotection. CD4⁺ T cells contribute to pain modulation by stimulating enteric neurons to release β‐endorphins, exerting an analgesic effect. In contrast, West Nile virus infection activates CD8⁺ T cells, which mediate neuronal death, while IFN‐γ upregulates MHC class I expression on enteric neurons, making them susceptible to CD8⁺ T‐cell‐induced damage. Additionally, B cells release IgE, which binds to FcεRI receptors on neurons, increasing intracellular calcium levels and promoting adenosine release. Adenosine then activates A3 receptors on mast cells, leading to their excessive activation. Mast cells also release tryptase, which acts on PAR2 receptors on dorsal root ganglion [DRG] neurons, activating PKCε and ultimately stimulating the TRPV1 channel, further modulating neuronal activity. This figure was created using Figdraw (www.figdraw.com).)

Beyond the gut, immune cells also play a crucial role in regulating neuronal function in the skin, influencing sensory perception, pain and itch. Mast cells release leukotriene C4 (LTC4), serotonin (5‐HT) and sphingosine‐1‐phosphate (S1P), which bind to Cysltr2, Htr1f and S1pr1 receptors on Nppb⁺DRG neurons, respectively. These interactions activate the gastrin‐releasing peptide (GRP) pathway, a critical mediator of itch sensation.^106^ In the pathology of atopic dermatitis, type 2 immune responses drive neuronal sensitisation. T helper 2 (TH2) cells,^107^ innate lymphoid cells type 2 (ILC2) and basophils^108^, ^109^ release IL‐4 and IL‐13, which act on IL‐4Rα/JAK1 signalling pathways in DRG neurons, leading to itch exacerbation.^109^ Thrombospondin‐1 (TSP‐1), a protein secreted by neutrophils and macrophages, binds to CD74 receptors on DRG neurons, inhibiting the PKA pathway and reducing TRPV1 sensitisation.^110^ The impact of immune cells on neuronal function is not limited to sensory perception, they also influence nerve regeneration and repair. Following sciatic nerve injury, CD8^+^ T cells are recruited to the damaged site, where they inhibit axonal regeneration by activating caspase 3, which in turn suppresses the pAKT and pS6 signalling pathways.^111^ The INDRA dataset (https://db.indra.bio) is a comprehensive platform that integrates potential interactions between neurons and immune cells, allowing for the prediction of neuroimmune interactions through specialised software. During the peak of pain (Tmax), the platform identified critical immune cell–nociceptor interactions, such as Ptgs2‐Ptgir,^112^ Hbegf‐Cd44^113^ and Aldh3a2‐Mrgprd,^114^ which are known to enhance nociceptor activity and promote pain or itch. These predictions help researchers identify genes and proteins that may influence pain amplification or resolution (Figure 2).

## 3 CROSSTALK BETWEEN NEUROIMMUNITY AND GUT MICROBIOTA

Numerous studies demonstrate neuroimmune–microbiota interactions in the context of inflammation. Following comprehensive research into the microbiota's interactions with other immune cells in IBD, current research is primarily focused on neuroimmunity.

### 3.1 Neuroimmunity affects the function of intestinal microbiota

**FIGURE 3:**
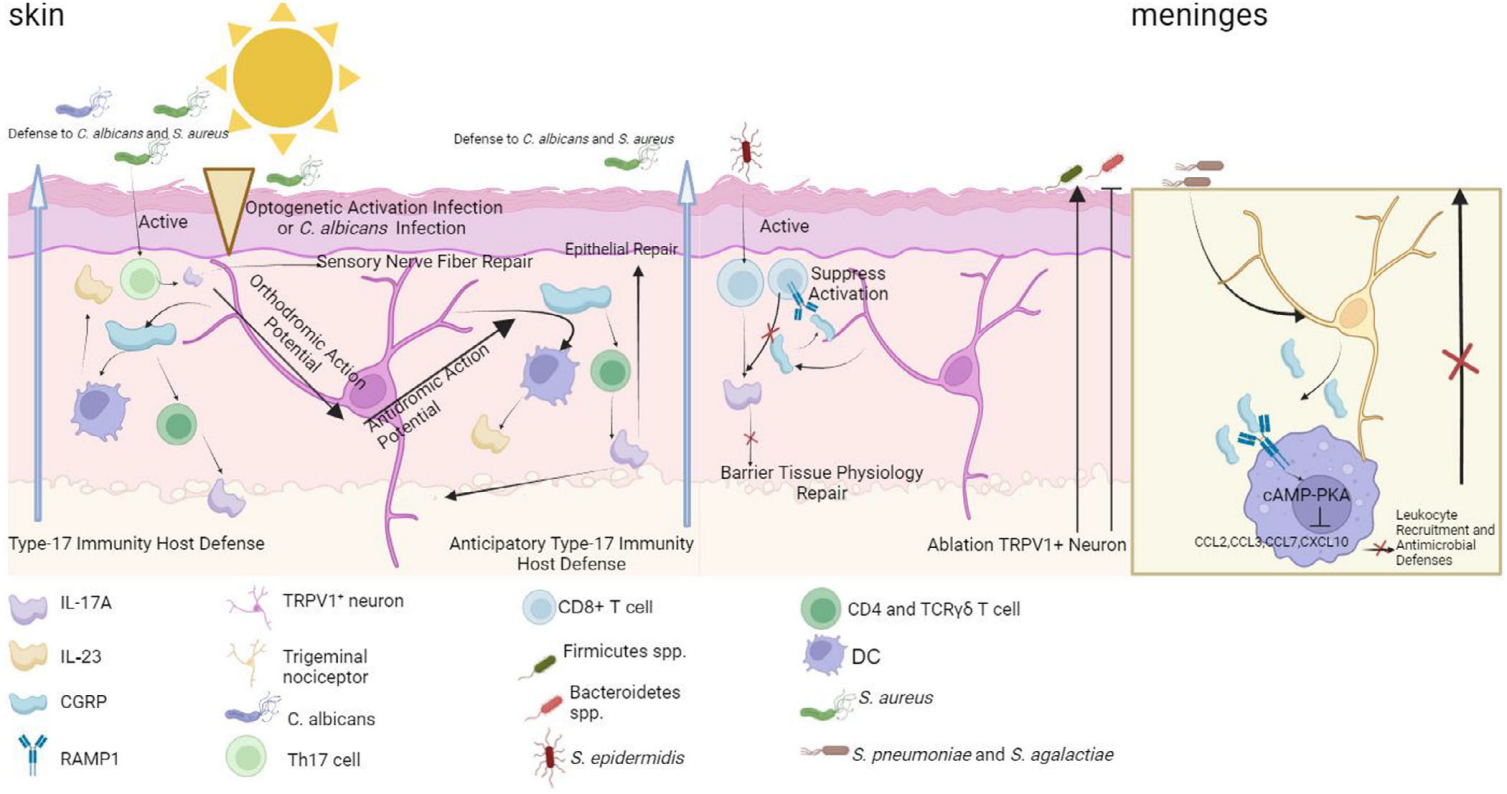
Neuroimmune regulation of gut microbiota function. (Optogenetic stimulation or *Candida albicans* infection activates TRPV1^+^ sensory neurons in the skin, leading to CGRP release, which in turn induces a Type‐17 immune response to counteract microbial invasion. Additionally, neurons can trigger anticipatory Type‐17 immune responses in adjacent areas through retrograde action potentials, exerting similar protective effects. *Staphylococcus epidermidis* stimulates CD8^+^ T cells, inducing a Type‐17 immune response. However, neurons in direct contact with these cells release CGRP, which binds to RAMP1 receptors on CD8^+^ T cells, suppressing the Type‐17 immune response. This inhibition promotes tissue repair and influences microbial invasion. In the meninges, *Streptococcus pneumoniae* and *S. agalactiae* activate trigeminal nociceptors, triggering the release of CGRP. CGRP, through its RAMP1 receptors on macrophages, activates the cAMP‐PKA pathway, leading to the suppression of neutrophil recruitment and antimicrobial activity. This figure was created using BioRender.com (https://biorender.com).)

Studies have revealed that neuroimmune interactions significantly influence the gut microbiota, affecting not only its composition but also its functionality. Neuroimmune signalling regulates the dynamic balance between the host and microbiota through intricate mechanisms. Specifically, sensory neurons establish direct communication pathways with the adaptive immune system via the release of neuropeptides such as CGRP, which modulate microbiota defence responses. For instance, optogenetic stimulation or *Candida albicans* infection can activate TRPV1^+^ sensory neurons, leading to the anterograde activation of neurons and the subsequent release of CGRP. CGRP, in turn, induces CD4^+^ T cells and TCRγδ T cells to secrete IL‐17A and stimulates dendritic cells to release IL‐23, thereby enhancing immunity against *C. albicans* and *Staphylococcus aureus*. Additionally, retrograde action potentials can stimulate neighbouring tissues, triggering type 17 immune responses and strengthening host defence mechanisms in surrounding areas.^115^ Furthermore, during *S. aureus* infection, Th17 cells directly interact with sensory nerve endings, leading to the release of IL‐17A, which binds to IL‐17RA receptors on sensory nerve fibres, promoting neuronal repair.^116^ Similarly, *Staphylococcus epidermidis* colonisation on the skin induces the accumulation of commensal‐reactive CD8^+^ T cells, which interact directly with sensory nerve terminals. In this context, CGRP released from sensory neurons binds to the RAMP1 receptor on T cells, inhibiting excessive CD8^+^ T cell activation and suppressing type 17 immune responses (IL‐17A and IL‐17F production). This mechanism promotes keratinocyte activation, ultimately regulating immune homeostasis in the skin.^117^ Moreover, CGRP, through its RAMP1 receptor on macrophages, activates the cAMP‐PKA pathway, inhibiting chemokine production and reducing neutrophil recruitment, thereby suppressing antibacterial defences.^118^ In these studies, nociceptor activation and CGRP release were shown to have dual functions: on one hand, activating CD4+ T cells to enhance type 17 immune responses, which protect against microbiota invasion and promote epithelial repair; on the other hand, suppressing CD8+ T cell activation, which modulates keratinocyte function to maintain skin barrier integrity. However, in the meninges, neuronal CGRP release has been found to suppress host antibacterial immunity rather than enhance it. This difference may arise due to the distinct immune environments of different tissues. While CGRP‐mediated immune activation in the skin is beneficial for infection clearance and tissue repair, excessive inflammation in the meninges could be detrimental, necessitating immune suppression in this region. These findings highlight the tissue‐specific role of CGRP in neuroimmune regulation (Figure 3). Meanwhile, neuroimmune signalling also impacts the metabolic functions of microbiota, as elucidated by metagenomics and metabolomics analyses. These functions include the production of short‐chain fatty acids (SCFAs), bile acid metabolism and vitamin synthesis. These metabolites not only modulate the host immune system but also influence the stability and functionality of the microbial community. For example, bile acid metabolism profoundly affects both immunity^119^ and the gut microbiota.^120^, ^121^ Furthermore, neuroimmune regulation of the gut microbiota exhibits regional specificity.^122^ In the proximal small intestine, it favours processes such as glucose absorption^123^ and dietary vitamin A metabolism.^124^ For instance, neuronal innervation of the upper small intestine is essential for the preabsorptive effects of rapamycin infusion in reducing glucose production.^125^ Importantly, the regulatory effects of neural signals on the gut microbiota are influenced by multiple factors, including diet, stress and disease‐related disruptions in barrier function. These findings emphasise the pivotal role of neuroimmune mechanisms in controlling gut microbiota functionality and maintaining host barrier homeostasis. They also provide crucial insights and foundations for further theoretical research and potential interventions targeting host‐microbiota interactions.

### 3.2 Intestinal microbiota regulates neuroimmune interactions

The microbiota affects the activity and function of resident immune cells and neurons by releasing a variety of bacterial components, metabolites and secreted factors. In IBD, adaptive and innate immune responses are dysregulated.^126^ Disruption of the mucus layer and disturbance of the intestinal epithelial barrier not only increases the direct contact of the microbiota with immune cells recruited to the epithelium, but also increases the activation of sensory neurons. The relationship between the gut microbiota and the neuroimmune system is essential for preventing chronic intestinal inflammation.

#### 3.2.1 Microbiota regulates peripheral sensory nervous function

In previous studies, the influence of the gut microbiome on organs beyond the gastrointestinal tract has led to the definition of several axes, including the ‘gut–liver axis’,^127^ ‘gut–brain axis’,^128^ ‘gut–muscle axis’,^129^ ‘gut–kidney axis’,^130^ ‘gut–lung axis’,^131^ ‘gut–heart axis’.^132^ Recently, a new concept of the gut‐peripheral nervous system axis has been proposed.^133^ The interaction between the gut microbiota and the peripheral sensory nervous system has garnered increasing attention. While gut epithelial and immune cells initiate protective responses when the intestinal barrier is disrupted, recent studies suggest that sensory neurons also participate in these defence processes.^134^ Traditionally, immune cell‐released inflammatory mediators during bacterial infections were considered the main triggers of nociception and pain activation. However, recent findings indicate that pathogens themselves can directly induce these responses.

**FIGURE 4:**
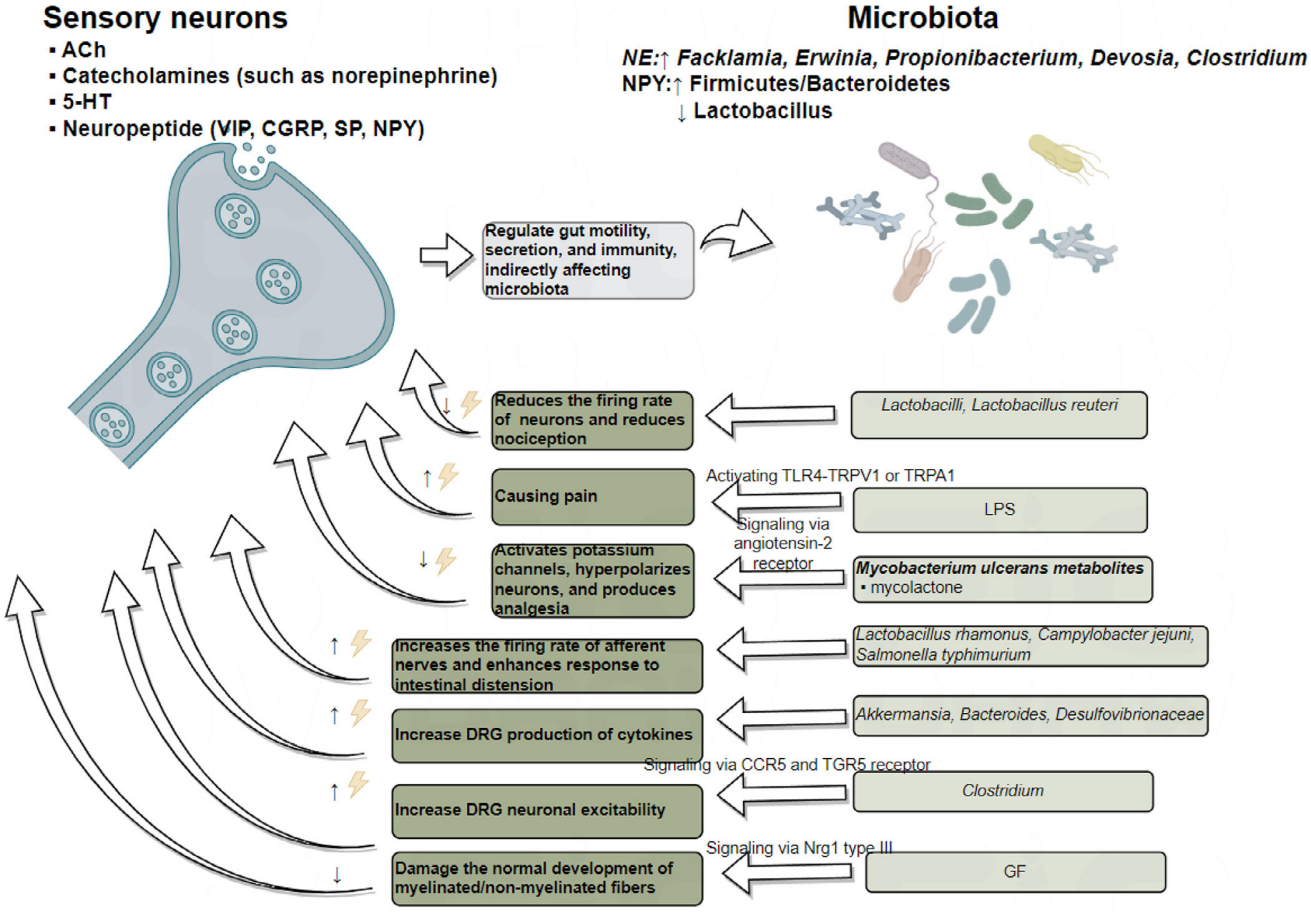
Crosstalk between sensory neurons and gut microbiota. (Sensory nerve endings in the gut regulate motility, secretion and immunity by releasing neuropeptides, acetylcholine, 5‐HT and catecholamines, maintaining gut microbiota homeostasis. Norepinephrine increases the abundance of *Facklamia*, *Erwinia*, *Propionibacterium*, *Devosia* and *Clostridium*, while neuropeptide Y [NPY] enhances the *Firmicutes/Bacteroidetes* ratio and reduces *Lactobacillus* levels. In response, microbes can influence neuronal firing. Additionally, lipopolysaccharide activates TRPA1 through TLR4 on neurons, triggering pain. Furthermore, microbial metabolites hyperpolarise neurons, producing analgesic effects. This picture was drawn by Figdraw (www.figdraw.com).)

Research has shown that germ‐free (GF) mice exhibit a lower density of enteric nerve fibres in the myenteric plexus, a reduced number of neurons, and a significant decrease in axon diameter. The gut microbiota impairs sensory neurons development in the DRG through the Nrg1 type III pathway.^133^ In the condition of gut microbiota depletion, thermal hyperalgesia or mechanical allodynia caused by chronic constriction injury, oxaliplatin or streptozocin treatment were suppressed, and cytokine production in the DRG was inhibited. Following faecal microbiota transplantation (FMT), neuropathic pain was restored, with *Akkermansia*, *Bacteroides* and *Desulfovibrionaceae* potentially playing a key role.^135^ The proliferation of *Clostridium* species leads to increased deoxycholic acid (DCA), which induces neuronal hyperexcitability through two mechanisms. First, DCA promotes immune cells to release CCL5, which acts on CCR5 receptors expressed on DRG neurons. Secondly, DCA directly binds to TGR5 receptors on DRG neurons. Both pathways contribute to neuronal hyperexcitability.^136^ Some microbes have analgesic effects. For example, *Lactobacillus* strains reduces the firing rate of lumbar DRG neurons and decreases nociceptive perception, particularly in response to colorectal distension.^137^ Additionally, metabolites of *Mycobacterium ulcerans*, such as mycolactone, activate Trek‐related potassium channels via angiotensin II receptor signalling, hyperpolarising neurons and producing analgesic effects.^138^ *Lactobacillus reuteri* also exhibits similar analgesic properties by reducing the firing rate of peripheral DRG neurons. LPS sensitises the TRPV1 channel via TLR4 and can also directly excite somatic and visceral nociceptive neurons through TRPA1.^139^ The activation of TRPA1 may result from GPCR‐mediated PLC activation, leading to PIP2 depletion.^140^. Gut pathogens like *Campylobacter jejuni* and *Salmonella typhimurium* can activate vagal sensory ganglia and solitary tract neurons, influencing pain perception.^141^ Moreover, *Lactobacillus rhamnosus* increases the firing rate of vagal afferent nerves, enhancing responses to gut distension.^142^ Figure 4 provides an overview of the mechanisms described above. Sensory nerve endings in the gut release neuropeptides, acetylcholine, 5‐HT and catecholamines, which regulate gut motility, secretion and immune responses, thereby maintaining the homeostasis of the gut microbiota. Conversely, the gut microbiota influences the activation of sensory neurons by releasing inflammatory factors, metabolites and other signalling molecules.

#### 3.2.2 Microbiota regulates immune cell function

**FIGURE 5:**
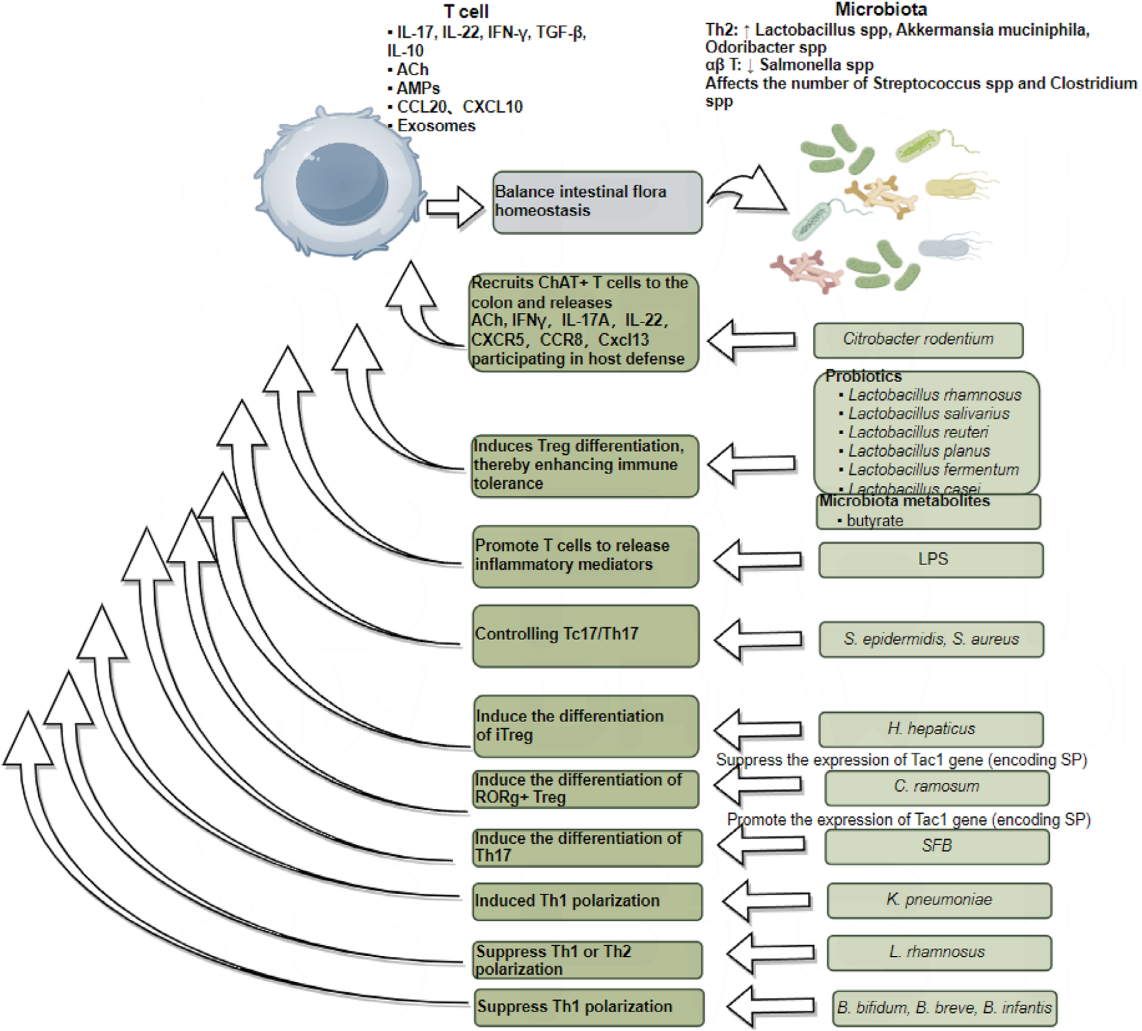
Crosstalk between T cells and gut microbiota. (In the gut, T cells maintain microbial homeostasis through inflammatory factors, acetylcholine, antimicrobial peptides [AMPs], chemokines and exosomes. Th2 cells promote the growth of beneficial bacteria such as *Lactobacillus* spp., *Akkermansia muciniphila* and *Odoribacter* spp., while αβ T cells reduce harmful *Salmonella* spp. abundance. Changes in T cells also affect the growth of *Streptococcus* spp. and *Clostridium* spp. In response, microbes can recruit T cells to the intestinal epithelium, influencing host defences by promoting T cell release of acetylcholine. Additionally, probiotics can mediate Treg cell differentiation, reducing inflammation and microbial metabolites like butyrate have a similar effect. Furthermore, Toll‐like receptor [TLR] ligand LPS influences immune regulation by promoting T cell release of inflammatory mediators. This picture was drawn by Figdraw (www.figdraw.com).)

The gut immune system comprises a large number of innate and adaptive immune cells, such as mast cells, macrophages, T cells and B cells. Different gut microbiota directly or indirectly influence immune responses by modulating these immune cells.^65^, ^143^ IBD arises from impaired immune tolerance to the gut microbiota, causing chronic inflammation.^144^ Early in life, the gut microbiota is crucial for establishing immune tolerance, particularly during the transition from breastfeeding to solid foods. This period is marked by the diversification of the gut microbiota and the withdrawal of immunoregulatory factors, such as maternal IgA from breast milk.^145^ Studies suggest that failure to establish gut tolerance during this critical window increases the risk of inflammatory diseases later in life.^146^ Furthermore, antigen‐presenting cells (APCs), such as Thetis Cell Group IV, which are key in inducing peripheral Tregs, emerge in the mesenteric lymph nodes in a developmental wave synchronised with weaning.^147^ These peripheral Tregs, in turn, establish the immunoregulatory tone within the gut. In IBD, the interactions between microbes and the host undergo significant changes, involving multiple immune pathways, such as the activation of Th17, Treg and B cells,^148^ the IL‐23/Th17 axis, autophagy regulation mechanisms,^149^ and alterations in Paneth cell function.^150^ The gut microbiota is essential for the differentiation and function of T cell subsets, such as Tregs and Th17 cells. For instance, during *Citrobacter rodentium* infection, the recruitment of ChAT^+^ T cells leads to the secretion of ACh, IFN‐γ, IL‐17A or IL‐22. Notably, CXCR5^151^ and CCR8^152^ expression is elevated, along with the chemokine CXCL13. CXCL13 plays a pivotal role in the formation of secondary lymphoid organs^153^, tertiary lymphoid tissues and the recruitment of IL‐22^+^ innate lymphoid cells (ILC3).^154^ Additionally, acetylcholine promotes the production of IL‐13 and IFN‐γ via M3 receptors on T cells, helping the host combat parasites like *Nippostrongylus brasiliensis* and *Salmonella typhimurium*.^155^ *Coriobacteriaceae ramosum* significantly suppresses the expression of the Tac1 gene, which encodes SP and other tachykinin neuropeptides.^156^ SP inhibits the differentiation of RORγ^+^ Tregs; thus, *C. ramosum* indirectly promotes RORγ^+^ Treg differentiation. In contrast, segmented filamentous bacteria (SFB) may enhance Tac1 expression via the SAA/IL‐23R pathway,^157^ thereby regulating SP levels. Given the antagonistic differentiation pathways of Tregs and Th17 cells, SFB promote Th17 cell differentiation. These findings suggest that microbial regulation of SP expression could be a critical early event in determining T cell differentiation.^93^ In IBD, DCs exhibit elevated CD40 expression,^158^ while T cells upregulate CD154 (CD40 ligand).^159^ Furthermore, CD4^+^ T cells producing IFN‐γ serve as a hallmark of the Th1 immune response in CD.^160^, ^161^ *Klebsiella pneumoniae* induces a robust Th1‐polarising response during DC maturation. In contrast, probiotic *L. rhamnosus* reduces cytokine production by DCs, thereby impairing their ability to polarise naive T cells into either Th1 or Th2 subsets.^162^ This results in a marked reduction of IFN‐γ and IL‐2 production by peripheral T cells in CD patients, potentially explaining the anti‐inflammatory effects of *L. rhamnosus*.^163^ Similarly, *Bifidobacterium bifidum*, *B. breve* and *B. infantis* stimulate macrophages to produce IL‐10 while downregulating IL‐12 and TNF‐α,^164^ thereby mitigating Th1 polarisation and alleviating mucosal inflammation in IBD. Additionally, Bifidobacterium modulates antigen presentation by reducing the proportion of DCs expressing CD80, which interacts with CD28 and CD152 (CTLA4) on T cells.^165^ The relative expression of CD80 and CD86 on APCs likely influences the type of T cell response generated. *B. breve* and *B. infantis* further decrease CD40 expression on DCs. CD40 signalling enhances IL‐12 production by DCs,^166^, ^167^ suggesting that probiotics may ameliorate IBD by disrupting DC‐T cell co‐stimulation through CD40/CD80 downregulation and IL‐10 upregulation, thereby suppressing Th1 polarisation. Research has also shown that *Staphylococcus epidermidis* and *S. aureus* induce Tc17 cells, which upregulate multiple genes associated with neuropeptides, neurotrophic factors or neuroendocrine signalling molecules (e.g., Ramp1, Ramp3, Ntrk3, Calca, Gch1, Nr3c1 and Bex3), suggesting a role for neural regulatory signalling in immune modulation.^117^ Meanwhile, *Helicobacter hepaticus* colonisation in the gut stimulates RORγt^+^ APCs, which, via CCR7, facilitate immune cell migration to specific tissues or lymph nodes. Additionally, integrin αvβ8 on the cell membrane activates TGF‐β, promoting iTreg differentiation, reducing inflammation and maintaining immune tolerance.^168^ Figure 5 summarises these interactions, illustrating how T cells regulate microbial abundance through inflammatory factors, acetylcholine and chemokines, while the microbiota reciprocally influences T cell activity through SCFAs and LPS. By interacting with the host immune system, these microbiota regulate immune responses and hold potential as therapeutic agents to alleviate inflammatory diseases such as IBD.

#### 3.2.3 Direct effects of intestinal microbial metabolites on neuroimmune function

**FIGURE 6:**
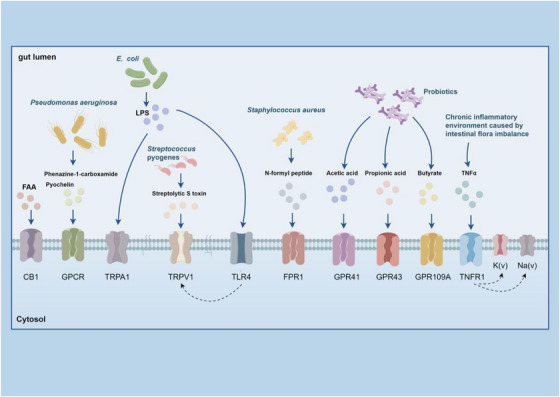
Gut microbiota affects neuronal activation by releasing various metabolites that bind to receptors on sensory neurons. (Gut microbiota releases various metabolites, including SCFAs, LPS and bacterial peptides, which bind to specific receptors on sensory neurons, such as CB1, GPCR, TRPA1 and TRPV1. These interactions modulate pain signalling. For example, butyrate influences neuronal activity via GPR109A, while LPS activates TRPA1 channels, sensitising nociceptive neurons. Highlighting the complex interplay between gut microbiota and the nervous system. This picture was drawn by Figdraw (www.figdraw.com).)

Gut microbiota metabolites, like SCFAs (acetate, propionate and butyrate), regulate immune functions by binding to specific receptors.^169^, ^170^ For example, butyrate induces Treg differentiation in mice, thereby enhancing immune tolerance.^171^ Microbial metabolites can also affect neuronal function through various pathways. For instance, fatty acid amide activates TRPV1^+^ DRG neurons via the CB1 receptor,^172^ and metabolites from *Pseudomonas aeruginosa*, such as phenazine‐1‐carboxamide and pyochelin, directly trigger G protein signalling pathways in chemosensory neurons.^173^ Additionally, microbial metabolites influence neuronal function through the aryl hydrocarbon receptor (AHR) on colonic enteric neurons,^174^ Although butyrate activates AHR in some cases,^135^ other studies indicate that it may inhibit ILC2‐dependent AHR activation,^175^ suggesting a controversy in the field. This discrepancy could be due to variations in experimental conditions, such as differences in microbial composition or immune environment, which may affect butyrate's interaction with AHR. Streptolysin S from *Streptococcus* can activate TRPV1^+^ neurons, causing pain responses. In response, these neurons release CGRP, which inhibits neutrophil recruitment, weakening the bactericidal response against *Streptococcus pyogenes*. *Streptococcus pneumoniae* activates Nav1.8^+^ nociceptive sensory neurons via its pore‐forming toxin pneumolysin, causing CGRP release from nerve endings. CGRP interacts with receptor activity modifying protein 1 on macrophages, altering their transcriptional, reducing chemokine expression, inhibiting neutrophil recruitment and reducing antimicrobial defences.^118^ However, some contradictory evidence suggests that pneumolysin activates neutrophils to release extracellular vesicles and form extracellular traps^176^ to combat microbial infections.^177^ These discrepancies further highlight the need for additional research to reconcile these findings. LPS derived from *E. coli* sensitises TRPV1 channels via a TLR4 mechanism.^178^ LPS also trigger nociceptive activity and pain via TRPA1^+^ neurons.^139^ However, emerging evidence suggests that LPS types vary among different Gram‐negative bacterial species, with some LPS molecules being less detectable by TLR4.^177^ Therefore, LPS‐induced neuronal activation may differ due to the concentration and structural heterogeneity of LPS molecules. During *S. aureus* infection, bacterial N‐formyl peptides binds to formyl peptide receptor 1, while the microbiota induces calcium flux and depolarisation in DRG neurons by forming α‐hemolysin pores on cell membranes, further leading to action potential firing.^179^ Butyrate can affect neuronal function via SCFA receptors (GPR41/FFAR3/GPR109A) expressed on enteric neurons, while acetylcholine mediates butyrate‐induced action potentials in submucosal neurons and myenteric neurons.^180^ *Lactobacillus acidophilus* induces the expression of μ‐opioid and cannabinoid receptors in intestinal epithelial cells, producing analgesic effects via the STAT3‐phosphorylation mechanism.^87^, ^181^ Additionally, *Lactobacillus murinus* and *Bacteroides fragilis* stimulate the excitability of intrinsic primary afferent neurons thereby regulating ENS activity.^182^ Some studies suggest that a reduction in *Bacteroidales* and *Erysipelotrichaceae* is associated with ENS recovery.^183^ In our previous study, we found that butyrate activates the PKC pathway on DRG neurons by promoting mast cell degranulation, leading to the upregulation of TRPV1 and aggravating visceral hypersensitivity.^184^ However, this finding contradicts other studies reporting that butyrate alleviates neuropathic pain^185^ by enhancing epithelial barrier function^186^ and reducing inflammation.^187^ These conflicting observations highlight the complexity of neuroimmune–microbiota interactions and underscore the need for further research to clarify the context‐dependent effects of butyrate on neuronal function and pain signalling. In conclusion, while considerable advancements has been made in understanding how microbial metabolites influence neuroimmune regulation, many issues remain unresolved. The conflicting findings further emphasise the complexity of these interactions. Figure 6 summarises the interaction between gut microbiota metabolites, including SCFAs, LPS and bacterial peptides, with receptors on sensory neurons. These interactions regulate pain signalling, highlighting the complex relationship between the gut microbiota and the nervous system.

## 4 NEUROIMMUNITY, MICROBIOTA AND IBD: MAKING CONNECTIONS

### 4.1 The relationship between gut microbiota and IBD

IBD is closely associated with changes in the gut microbiota's composition and function. Research has shown that the gut microbiota of IBD patients is significantly imbalanced,^188^, ^189^ marked by a decline in beneficial bacteria, like *Faecalibacterium prausnitzii* and *Roseburia* (which promote SCFA production and Treg cell differentiation), and a rise in harmful bacteria, like *Enterobacteriaceae* (*E. coli*/*Shigella*, which activate inflammatory cascades via TLR4 signalling). This dysbiosis is closely linked to various clinical symptoms of IBD, such as abdominal pain, bloating and diarrhoea. The deficiency of specific beneficial bacteria and the overgrowth of harmful bacteria aggravate intestinal inflammation and trigger a range of associated symptoms through neuroimmune mechanisms. The reduction of SCFA‐producing bacteria like *Roseburia* and *Faecalibacterium* not only decreases butyrate levels, impairing epithelial barrier regulation, but also limits anti‐inflammatory effects. Concurrently, the proliferation of inflammatory bacteria such as *Clostridium* exacerbates the immune response, leading to more severe symptoms (Table 2).^190^ Past research primarily focused on bacteria with differential abundance, but the latest study, by analysing evolutionary signals in the human gut microbiome, uncovered hundreds of strains associated with IBD. Among these, strains of *E. lenta* were found to have a negative correlation with IBD activity, potentially playing a protective role. The study also identified genetic differences in these strains related to oxidative stress, nutrient synthesis, antibiotic resistance and cell wall pathways. For instance, strains containing potential virulence factors like adhesins and hemolysins may contribute to persistent inflammation in IBD. These findings suggest that these strains are adapted to conditions associated with IBD, such as immune responses and metabolic changes, driving the progression of the disease.^191^ A non‐invasive diagnostic tool based on multiplex digital PCR has been developed, which is a highly sensitive detection technique capable of quantifying specific bacteria. This tool aims to facilitate early diagnosis and management of IBD by effectively distinguishing between IBD patients and control subjects.^192^ In small phase II clinical trials, FMT has been shown to achieve remission in approximately 30% of patients with UC. Significant progress has been made in phase III clinical trials and drug approvals. In the treatment of CD with FMT, antibiotic preconditioning and multiple administrations are the main factors influencing success rates. The success of microbiome therapy in IBD depends on precisely controlled dosing, standardised components and appropriate preconditioning measures. These factors can ensure that more patients benefit from the treatment while advancing the therapy towards broader clinical applications.^193^

**TABLE 2:** Dysbiosis in the gut microbiota of inflammatory bowel disease (IBD) patients and functional analysis.

Microbiota	Crohn's disease (CD)	Ulcerative colitis (UC)	Related functions/significance	Refs.
*Firmicutes*	Decreased	Decreased	Indicates overall decrease in biodiversity	^247^
*Proteobacteria*	Increased	Increased	Common in patients with intestinal inflammation	^247^
*Roseburia*	Decreased	Decreased	Increases Treg cells and butyrate levels	^248^
*Phascolarctobacterium*	Decreased	Decreased	Increases propionate	^248^
*Clostridium*	Increased	Increased	Possibly associated with intestinal inflammation	^248^
*Ruminococcaceae*	Decreased	–	Acetate‐producing microbiota	^248^
*Leuconostocaceae*	–	Decreased	Acetate and lactate‐producing microbiota	^248^
*Enterobacteriaceae* *(E. coli/Shigella)*	Increased	–	Activates TLR4 signalling pathway, triggering inflammatory cascades	^248^
*Faecalibacterium*	Decreased	Decreased	Host epithelial repair and inflammation regulation	^248^
*odoribacterium*	Decreased	Decreased	Host epithelial repair and inflammation regulation	^248^
*Faecalibacterium prausnitzii*	Decreased	Decreased	Produces butyrate, which has anti‐inflammatory effects in IBD	^248^

Around half of IBD patients develop at least one extra‐intestinal manifestation,^194^ with common systems affected including the musculoskeletal, skin, ocular, hepatobiliary and oral systems.^195^ Additionally, IBD patients are prone to neuropsychiatric conditions like depression and anxiety. Numerous studies have identified comparable patterns of gut microbiota dysbiosis in IBD and different extra‐intestinal manifestation, suggesting that gut microbiota plays a significant role in the pathophysiology of these conditions. Specifically, the gut microbiota contributes to the onset of IBD and related extra‐intestinal manifestation through several mechanisms: impaired gut barrier function, microbial translocation, molecular mimicry, microbiota‐related metabolites and immune cell activation.^195^ Moreover, the gut microbiota plays a crucial role in the anxiety‐like behaviour associated with colitis via the gut‐brain axis.^135^ It has also been reported that metal polyphenol‐based antidepressants can alleviate colitis‐induced neuropsychiatric disorders through the microbiota–gut–brain axis by reducing neuroinflammation, enhancing hippocampal neuroplasticity, regulating hippocampal immune responses and restoring neurotransmitter homeostasis.^196^ Thus, while the mechanisms through which the gut microbiota mediates IBD and its associated neurological diseases share similarities, particularly in immune modulation and inflammation, there are notable differences. Both IBD and neurological diseases involve gut microbiota‐driven immune activation and inflammation, but the pathophysiology of neurological diseases is more dependent on specific mechanisms involving the gut‐brain axis. Future research are expected to reveal more complex interactions between the nervous system and the gut microbiota, which may offer new therapeutic strategies, including probiotics,^109^, ^197^, ^198^, ^199^, ^200^, ^201^ prebiotics,^202^, ^203^ postbiotics,^204^, ^205^, ^206^ antibiotics,^207^ and FMT.^208^ These approaches not only hold promise for improving the prognosis of extra‐intestinal manifestation patients but also offer the potential for alleviating neurological symptoms.

### 4.2 Neuroimmunity is the intersection between IBD and gut microbiota

In a study of 134 genes associated with IBD (data source: Alliance of Genome Resources), many of these genes are related to neuroimmune functions that involve mechanisms of microbial defence. For example, they include humoral immunity (*CLDN1* and *NOS3*), cytokine production (*IL33/MIR21/EPHB2*) and response to LPS (*FOS*). This underscores the importance of immune responses in disease progression. Several genes that influence neuroimmune cell functions are associated with IBD, such as *BDNF* ^209^ and *GDNF*.^210^ These genes play crucial roles in regulating neuronal death and are involved in guiding axon and neuron projections, as well as axon development and maturation. *CRHR2* is involved in neuroactive ligand–receptor interactions,^211^ while *CRHR1* is involved in shaping synaptic development and neuronal maturation.^212^ Additionally, genes like *IL33* ^213^ and *EPHB2* ^214^ are implicated in the positive regulation of leukocyte transport and activation. Specifically, *MIR21* regulates cellular responses after stimulation of T cell receptors.^215^ The protein NEMO/IKKγ encoded by *IKBKG* is essential for activating the NF‐κB pathway and participates in various physiological and cellular functions, including immunity.^216^ These results emphasise the essential role of neuroimmune functions in IBD progression. Gut microbiota influences IBD through neuroimmune. For instance, gut microbiota can promote ENS neurons to release IL‐6, which subsequently influences T cell differentiation. A moderate dose of IL‐6 drives T cell differentiation into RORγ⁺ Tregs, which have protective effects against IBD.^89^ Similarly, *Clostridium ramosum* suppresses TRPV1⁺ DRG neurons from releasing SP,^93^ thereby facilitating RORγ⁺ Treg differentiation.^66^ Studies have shown that this subset of Tregs plays a protective role in IBD mouse models.^66^, ^67^, ^68^ Moreover, *Y. pseudotuberculosis* stimulates sympathetic neurons to release NE, which acts on β₂‐ARs on macrophages. This activation induces macrophages to release Arg1, which prevents neuronal loss and motility disorders, thereby preserving ENS integrity and function—a beneficial effect for IBD.^96^ However, contradictory evidence exists; one study found that mice with haematopoietic and endothelial cell‐specific Arg1 deficiency exhibited faster recovery from colitis.^217^ This discrepancy could be attributed to differential Arg1 activity in various immune and neuronal compartments, highlighting the complexity of neuroimmune regulation in IBD. Additionally, *C. rodentium* has been implicated in IBD pathogenesis,^218^, ^219^ though its precise mechanism remains unclear. It has been shown that *C. rodentium* recruits Chat⁺ T cells, which release IFN‐γ,^154^ IFN‐γ, in turn, induces enteric neurons to express MHC I, leading to CD8⁺ T cell recruitment and subsequent neuronal damage.^21^ This mechanism may contribute to IBD progression by disrupting neural‐immune homeostasis in the gut. *L. rhamnosus* has been reported to ameliorate localised inflammation in DSS‐induced colitis mouse models.^220^ On one hand, it exerts anti‐inflammatory effects by inhibiting T cell differentiation into Th1 or Th2 subsets.^162^, ^163^ However, on the other hand, *L. rhamnosus* enhances vagal afferent nerve firing and increases responses to gut distension, which could exacerbate IBD‐related visceral hypersensitivity.^142^ Conversely, *C. albicans* worsens IBD,^220^ likely due to its ability to stimulate TRPV1⁺ neurons to release CGRP, which acts on CD4⁺ T cells to promote IL‐17A production. Since Th17 cell expansion is strongly linked to inflammatory disease susceptibility,^60^ and is markedly elevated in IBD,^61^ this mechanism suggests a pathogenic role for *C. albicans* in disease exacerbation. Similarly, *Akkermansia muciniphila* promotes CD4^+^ T cell differentiation into Tregs, increases SCFA production, and downregulates pro‐inflammatory cytokines, thereby alleviating inflammation. Moreover, environmental factors, such as conventional animal facility influence gut microbiota composition, potentially exacerbating T cell‐dependent IBD symptoms, as detailed in Table 3. These findings highlight the neuroimmune mechanisms through which gut microbiota modulates disease progression in IBD. These data support the association between neuroimmune functions, changes in gut microbiota and the development of IBD.

**TABLE 3:** The mechanism and impact of intestinal microbiota affecting inflammatory bowel disease (IBD) through neuroimmunity.

Intervention	Mechanism	Impact on IBD	Refs.
**Antibiotic and drug interventions**			
Antibiotic mixture (Abx) + *Clostridium butyricum*	Reduces Th1, Th17; increases Th2 cells and SCFA production	Improves inflammation and barrier damage, alleviates IBD symptoms	^249^
Vancomycin	Polarises CD4+ T cells towards Th1/Th17, dorsal root ganglion (DRG) neurons are overexcited	Worsens IBD by exacerbating inflammation	^250^, ^251^
Metronidazole	Induces iNKT cells to polarise towards IL‐10 production	Improves IBD by reducing inflammation	^250^
Isotretinoin	Induces IL‐10 signalling in Treg and naïve T cells	Improves IBD by reducing inflammation	^252^
Streptomycin	Induces pro‐inflammatory expression profile	Worsens IBD	^252^
BTK	Modulates T cell Th1 polarisation	Reduces the incidence and severity of IBD inflammation	^253^
**Probiotic interventions**			
*Lactobacillus rhamnosus*	Improves gut microbiota and SCFA levels; increases Treg proportion	Alleviates IBD‐associated hypermotility and pain	^180^
*Limosilactobacillus reuteri*	Increases Foxp3+CD4+ T cells; reduces pro‐inflammatory cytokines; increases tight junction proteins and HSP70, HSP25	Improves IBD by enhancing intestinal barrier function	^254^
*Akkermansia muciniphila*	Regulates CD4+ T cell differentiation into Tregs; increases short‐chain fatty acid production; downregulates pro‐inflammatory cytokines	Alleviates chronic IBD	^255^
*Faecalibacterium*	Promotes Treg secretion of IL‐10	Prevents and alleviates IBD; reduces chronic intestinal inflammation	^256^
Se–Se embedded *Lactobacillus casei*	Improves gut microbiota and SCFA levels; increases Treg proportion	Improves IBD by mitigating inflammation and enhancing gut barrier function	^257^
**Plant‐derived and natural interventions**			
Tieguanyin oolong tea polysaccharides	Modulates T cells; adjusts gut microbiota	Improves IBD by mitigating inflammation and enhancing gut barrier function	^258^
*Chlorella*	Alters gut microbiota and SCFA composition; increases Treg levels	Improves IBD by alleviating inflammation and tissue damage	^259^
Naticol®Gut	Modulates CD4+ T cell enhancement of Th2 response; inhibits CD8+ T cell activation; balances gut microbiota	Improves IBD by alleviating pathological symptoms such as colon shortening, inflammation and tissue damage	^260^
Sulphate‐reducing bacteria peptides	Decreases IL‐17A from γδ T cells, ILC3s, Th17; prevents naïve CD4+ T cell differentiation into Th17; reverses dysbiosis	Improves DSS‐induced IBD by reducing gut inflammation	^261^
**Environmental factors**			
Environmental factors (conventional animal facility, non‐SPF)	Influences gut microbiota composition, increases circulating inflammatory cytokines as well as Th1 and Th17 cells	Promotes IBD by heightened inflammation and greater intestinal barrier dysfunction	^262^
**Bacterial and other microbial interventions**			
Gram‐negative bacteria	Reduces calcium‐dependent potassium channel opening; decreases sAHP in sensory AH neurons	Contributes to visceral pain and neurogenic inflammation in IBD	^139^
*Citrobacter rodentium*	Increases excitability of colonic DRG neurons; ChAT+ T cells recruited to colon	Contributes to visceral hyperalgesia in IBD	^263^
Dectin‐1	Sensitises TRPV1 + DRG neurons	Enhances visceral pain sensitivity and inflammation in IBD	^192^

### 4.3 Next‐generation technologies for exploring neuroimmune interactions

The rapid development of next‐generation sequencing technologies like single‐cell RNA sequencing (scRNA‐seq) and single‐nucleus RNA sequencing (snRNA‐seq) has led to significant advancements in understanding the neuroimmune landscape of the gut. These technologies allow for high‐resolution analysis of the complex interactions between gut immune cells, neurons and microbial communities. For example, scRNA‐seq has revealed the unique role of FOXP3^+^ Tregs in IBD, particularly their involvement in immune tolerance and inflammatory responses.^184^ Furthermore, studies have shown that the absence of the AHR repressor in intraepithelial lymphocytes increases susceptibility to *Clostridium difficile* infection and dextran sulphate sodium‐induced colitis, mechanisms that are closely linked to oxidative stress and ferroptosis.^221^ In addition, scRNA‐seq studies have uncovered interactions between *Bacteroidales* and *Clostridiales* bacteria with IL1B^+^ myeloid cells in both pouchitis and ulcerative colitis, identifying FOXP3/BATF^+^ T cells as key players in immune dysregulation. These findings offer valuable insights into the inflammatory mechanisms of IBD.^222^ Additionally, microbiome studies have demonstrated that metabolites such as succinate produced by gut microbes can promote tuft cell expansion, thereby alleviating ileal inflammation in mouse models of CD. The expansion of tuft cells was associated with an increase in GATA3^+^ cells and type 2 cytokines (IL‐22, IL‐25, IL‐13), while reducing RORγt^+^ cells and type 17 cytokines (IL‐23), thus modulating the immune response.^223^ Moreover, snRNA‐seq sequencing technology provides us with a comprehensive map of the close interactions between the microbiota and various cell types.^224^ Through these technologies, we can not only uncover how the gut microbiota influences the pathogenesis of IBD through the neuroimmune network, but also potentially identify new biomarkers and therapeutic targets for future IBD treatments.

In summary, single‐cell and single‐nucleus RNA sequencing technologies have provided invaluable tools for unravelling the complex relationships between gut microbiota, neuroimmune functions and IBD. In the future, these technologies may pave the way for the identification of new biomarkers and precision therapies in the management of IBD.

## 5 POTENTIAL THERAPEUTICS TARGETING DISRUPTED NEUROIMMUNE INTERACTIONS AND MICROBIOTA DYSREGULATION

The association between neuroimmune dysregulation and various diseases, for example, in neuropsychiatric disorders such as schizophrenia,^225^ suggests the need to further explore the regulation of neuroimmune interactions and their therapeutic potential. Therapeutics for this disorder are continuing to develop and show significant clinical promise. First of all, VGX‐1027, as an effective immune modulator, can significantly reduce pro‐inflammatory cytokines (e.g., 6, TNF‐α and IFN‐γ) expression, while elevating the levels of the anti‐inflammatory cytokine IL‐10. This modulation not only restored neuroimmune balance, but also reduced neuroinflammation and improved social deficits in a BTBR mouse model, showing its potential to alleviate autism‐like symptoms caused by immune dysregulation.^226^ Secondly, imipramine regulates the cAMP/PKA pathway by inhibiting the hypothalamic‐pituitary‐adrenal axis and sympathetic nervous system, reducing the levels of IL‐6 and bone marrow precursor cells. This mechanism not only effectively blocks the dysregulation of neuroimmune interactions, but also has anxiolytic and antidepressant effects.^227^ In the study of neuroimmune regulation, bioelectronic medicine, as an emerging field, emphasises the important role of the vagus nerve in regulating peripheral immune function. Electrical vagus nerve stimulation has shown significant therapeutic potential in preclinical models of IBD.^228^ Other technologies such as deep brain stimulation and transcranial magnetic stimulation^147^, ^229^, ^230^ are also actively exploring their clinical applications. Furthermore, studies have used biomaterials and tissue engineering techniques to modulate immune responses and enhance nerve regeneration.^231^ Engineered immunomodulatory strategies include scaffold‐based technologies^232^ and cell therapies^233^ that improve the regenerative effects of nerve grafts by modulating immune responses. For example, implanting nerve guidance channels containing IL‐4^234^ or collagen VI^235^ into rat models effectively modulates macrophage phenotypes and promotes axonal regeneration. A new strategy has been developed for the first time using a biomaterial platform to enable drug‐containing microparticles to induce and maintain an anti‐inflammatory macrophage phenotype in an inflammatory environment, which may promote nerve regeneration and maintenance by regulating macrophage behaviour, thereby contributing to functional restoration.^236^ In addition to macrophages,^237^ nanomaterials targeting T cells^238^, ^239^ and neutrophils^240^, ^241^ may provide new strategies to promote nerve regeneration through immunomodulatory therapies.

Dysbiosis can lead to dysregulated neuroimmune interactions.^242^ In studies of gut microbiota dysbiosis, *mucosal fungi* may modulate mouse behaviour through type 17 immune mechanisms.^243^ The newly proposed liver–brain–enteric neural arc mechanism suggests that hepatic vagal sensory nerves can transmit intestinal microenvironmental signals to regulate enteric neurons, a process that helps maintain intestinal immune homeostasis.^244^ Specific probiotics, such as *Lactobacilli*, have been shown to be effective in treating diarrhea^245^, ^246^ and produce analgesic effects by modulating receptor expression in the intestinal mucosa.^87^

In summary, therapeutic strategies targeting specific microbial–neuroimmune interactions may offer novel treatments for conditions such as IBD and other gastrointestinal infections, thereby bringing new hope to clinical practice.

## 6 PERSPECTIVES AND CONCLUSIONS

Although neuroimmune interactions play a central role in inflammatory diseases such as IBD, several challenges hinder the development of therapeutic strategies targeting this axis. Neuroimmune research is inherently complex. This is due to the highly dynamic nature of interactions between the nervous and immune systems, which vary over time and space. For instance, the regulatory mechanisms of the nervous system differ between acute and chronic inflammation, and these controls are dependent on the local microenvironment. Therefore, accurately understanding how these interactions change over time and location requires advanced techniques and sophisticated data integration. Moreover, neuroimmune interactions often occur in highly specialised tissues or organs, such as the brain or gut, and many in vitro or mouse models may not fully replicate these complex interactions in the human physiological environment. There is an urgent need to develop technologies that allow dynamic spatiotemporal research, such as spatial transcriptomics, single‐cell sequencing organoid models and multi‐omics integration, to fully unravel the dynamic mechanisms and regulatory networks of neuroimmune cell interactions in health and disease.

Targeting gut microbiota to modulate the functions of neuroimmune cells, alongside other therapies, may offer a promising approach for treating IBD. Different types of symbiotic and pathogenic bacteria can regulate the activity of sensory neurons and the immune cells with which they interact. However, clinical trials for such therapies face significant challenges, particularly when dietary interventions involve various biases and uncontrolled factors. In this regard live biotherapeutic products represent an exciting alternative. Live biotherapeutic products are novel biologics containing live microorganisms designed for the prevention or treatment of human diseases. Increasing attention is being given to identifying bacterial strains that can manage chronic diseases.

Therefore, clarifying how different sensory neuron subtypes interact with both the innate and adaptive immune system is crucial for understanding their roles in maintaining homeostasis, mediating inflammation and defending the host. Ultimately, targeting these specific microbial–neuroimmune interactions could provide novel therapeutic approaches for conditions such as IBD.

## AUTHOR CONTRIBUTIONS

Jinxia Zhai proposed the idea and wrote the original manuscript. Cong Dai, Yingjie Li and Jiameng Liu edited the manuscript. All the authors approved the final version of the manuscript.

## CONFLICT OF INTEREST STATEMENT

The authors declare no conflicts of interest.

## ETHICS STATEMENT

Not applicable.

## Data Availability

No data were used for the research described in the article.
